# The genome of medicinal leech (*Whitmania pigra*) and comparative genomic study for exploration of bioactive ingredients

**DOI:** 10.1186/s12864-022-08290-5

**Published:** 2022-01-24

**Authors:** Lei Tong, Shao-Xing Dai, De-Jun Kong, Peng-Peng Yang, Xin Tong, Xiang-Rong Tong, Xiao-Xu Bi, Yuan Su, Yu-Qi Zhao, Zi-Chao Liu

**Affiliations:** 1 Engineering Research Center for Exploitation and Utilization of Leech Resources in Universities of Yunnan Province, School of Agronomy and Life Sciences, Kunming University, Kunming, 650214 China; 2 State Key Laboratory of Primate Biomedicine Research; Institute of Primate Translational Medicine, Kunming University of Science and Technology, Kunming, 650500 China; 3 Department of Integrative Biology and Physiology, University of California, Los Angeles, Los Angeles, CA 90095 USA

**Keywords:** *Whitmania pigra*, Genome, Bioactive Ingredients, *Helobdella robusta*, *Hirudo medicinalis*

## Abstract

**Background:**

Leeches are classic annelids that have a huge diversity and are closely related to people, especially medicinal leeches. Medicinal leeches have been widely utilized in medicine based on the pharmacological activities of their bioactive ingredients. Comparative genomic study of these leeches enables us to understand the difference among medicinal leeches and other leeches and facilitates the discovery of bioactive ingredients.

**Results:**

In this study, we reported the genome of *Whitmania pigra* and compared it with *Hirudo medicinalis* and *Helobdella robusta*. The assembled genome size of *W. pigra* is 177 Mbp, close to the estimated genome size. Approximately about 23% of the genome was repetitive. A total of 26,743 protein-coding genes were subsequently predicted. *W. pigra* have 12346 (46%) and 10295 (38%) orthologous genes with *H. medicinalis* and *H. robusta*, respectively. About 20 and 24% genes in *W. pigra* showed syntenic arrangement with *H. medicinalis* and *H. robusta*, respectively*,* revealed by gene synteny analysis. Furthermore, *W. pigra, H. medicinalis* and *H. robusta* expanded different gene families enriched in different biological processes. By inspecting genome distribution and gene structure of hirudin, we identified a new hirudin gene g17108 (hirudin_2) with different cysteine patterns. Finally, we systematically explored and compared the active substances in the genomes of three leech species. The results showed that *W. pigra* and *H. medicinalis* exceed *H. robusta* in both kinds and gene number of active molecules.

**Conclusions:**

This study reported the genome of *W. pigra* and compared it with other two leeches, which provides an important genome resource and new insight into the exploration and development of bioactive molecules of medicinal leeches.

**Supplementary Information:**

The online version contains supplementary material available at 10.1186/s12864-022-08290-5.

## Background

Leeches are segmented parasitic or predatory worms that belong to the phylum Annelida and the subclass Hirudinea with the ability to extend or contract their bodies [1–3]. Most leeches live in freshwater environments, while some species can be found in terrestrial and marine environments. The best-known leeches, such as European medicinal leech *Hirudo medicinalis,* are hematophagous, feeding on vertebrate blood and invertebrate hemolymph [4–6]. *H. medicinalis* attaches to the host by means of its two suckers and bites through the skin of its victim. Most leech species, however, are predatory, feeding primarily by swallowing other invertebrates. Almost 700 species of leeches are currently recognized, of which some 100 are marine species, 90 terrestrial and the remainder freshwater taxa.

Although there are a huge diversity and a close relationship to people, we know little about the genome of leeches. In 2013, one leech *H. robusta* was sequenced to study bilaterian evolution [7]. *H. robusta* is a freshwater leech in the family *Glossiphoniidae*, and a type of annelid with anterior and posterior suckers that are used for locomotion and feeding on blood. Its early development has been studied extensively. For another important family *Hirudinidae*, the genome of *H. medicinalis* has been reported recently and studied from different perspectives by Babenko VV [8] and Kvist S [9], respectively. The family *Hirudinidae* includes medicinal leeches which have been widely utilized in medical procedures for thousands of years. Because of their important bioactive ingredients, medicinal leeches, such as *H. medicinalis* and related species, have engendered great interest from pharmaceutical companies.

**Fig. 1:**
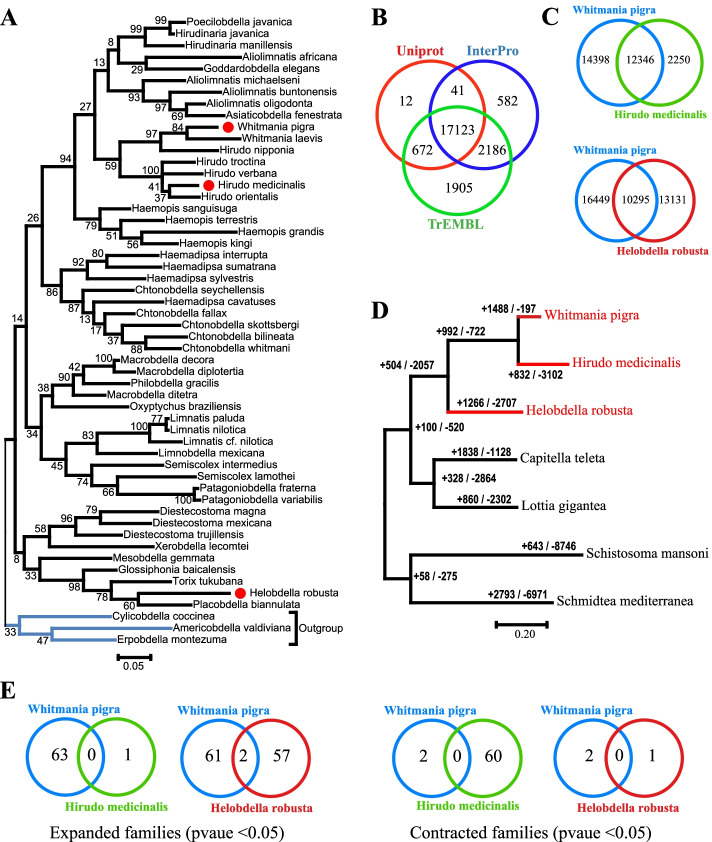
Genome annotation and evolution of *W. pigra* compared with *H. robusta* and *H. medicinalis*. **A** Phylogenetic analysis of leech species by Maximum Likelihood method based on COI genes. Highlighted with red dots correspond to three leech species compared with in our study. Posterior probabilities are assigned to the node; **B** the predicted protein-coding genes with matching entries in the three popular public databases; **C** the Venn diagram showed the orthologous genes between *W. pigra* and *H. medicinalis* (top panel), and between *W. pigra* and *H. robusta* (bottom panel); **D** Gene expansion and contraction in the *W. pigra* genome. The number of expanded (+) and contracted (-) gene families are shown along branches and nodes. **E** the Venn diagram showed the number of expanded and contracted gene families between *W. pigra* and *H. medicinalis,* and between *W. pigra* and *H. robusta*

Comparative study of these available genomes of leeches facilitates the discovery of bioactive ingredients. In this study, we reported the genome of another medicinal leech *W. pigra* in the family *Hirudinidae* and compared it with other two leech species (Fig. 1A). *W. pigra*, an Asian freshwater leech, is non-blood feeding, despite the placement of this genus within the family *Hirudinidae* [10]. The family *Hirudinidae* also includes *H. medicinalis* and several other blood feeding species. *W. pigra* is a macrophagous leech and it commonly swallows or takes bites out of prey sources [11–13]. According to the current Chinese Pharmacopoeia*, W. pigra,* as a source of medicinal leeches, is the most commonly available from the Chinese commercial leech market [14]. We first analyzed the genome of *W. pigra* and conducted gene synteny analysis among the three leech species *H. robusta*, *W. pigra, and H. medicinalis.* Then we analyzed the expansion and contraction of gene family among seven related species (*H. robusta*, *Lottia gigantea*, *Capitella teleta*, *Schmidtea mediterranea*, *Schistosoma mansoni*, *W. pigra, H. medicinalis*). The sequence diversity, genome distribution and gene structure of hirudin were also studied. At last, we explored nine kinds of bioactive compounds in the genomes of the three leech species. This study pointed out the differences in the genome of the three leech species *W. pigra, H. medicinalis* and *H. robusta,* and provided insight into the exploration and development of the bioactive molecules of medicinal leeches.

## Results

### Summary of genome assembly and annotation for *W. pigra*

Using a whole-genome shotgun strategy with the Illumina HiSeq™ 2000 platform, we sequenced the genome of *W. pigra* from Wuhan, the provincial capital Hubei, China. The de novo assembly of a 146 Gbp high-quality sequences from 2 paired-end and 3 mate-pair libraries provided 100-fold coverage with a total assembly length of 177 Mbp (Table 1), which approximates the genome size estimated by 23 K-mer distribution (Fig. S1). The scaffold N50 is 728 Kbp. 3495 scaffolds are with length >2 Kbp. Repeat content comprised 23% of the *W. pigra* genome, which is 10% lower than that of the *H. robusta* [7]. The *W. pigra* shares a similar profile of GC content (35%) with *H. robusta* (33%), lower than that of *H. medicinalis* (41%). A total of 26,743 protein-coding genes were predicted in *W. pigra*. *W. pigra* and *H. Robusta* showed similar gene model features in a whole. However, *W. pigra* has shorter intron length and longer protein length compared with *H. robusta* (Table 1). A total of 17123 protein-coding genes were annotated in all three common databases Uniprot, TrEMBL and interPro (Fig. 1B). We identified 12346 and 10295 orthologous genes between *W. pigra* and *H. medicinalis*, and between *W. pigra* and *H. robusta*, respectively*,* using the reciprocal best blast hits (RBHs) method (Fig. 1C). There are a large proportion of genes (14398 and 16449) in *W. pigra* not assigned as orthologous genes.

**Table 1:** Summary for genome sequencing, assembly and annotation

	*H. robusta* (Ref [7])	*W. pigra* (this study)	*H. medicinalis* (Ref [8])	*H. medicinalis* (Ref [9])
Size of genome assembly	228 Mbp	177 Mbp	187Mbp	177Mbp
Num. of Scaffolds	1,993	10,050	14,042	19,929
Num. of scaffolds (> 2Kbp)	1124	3495	5277	10128
Scaffold N50	3,060 Kbp	728 Kbp	97 Kbp	504Kbp
Total reads	3,176,156	118,388,619	62,184,084	NA
Reads mapping to genome (%)	2,839,951 (89%)	112,480,685 (95%)	NA	NA
Sequencing coverage depth	7.92X	100X	73X	146X
Repetitive content (%)	33	23	NA	24
GC (%)	33	35	41	35
Num. of predicted genes	23,400 ^**a**^	26,743 ^**a**^	14,596 ^**a**^	17205 ^**a**^
Protein length	376	438	464	NA
Mean exon length	203 bp	205 bp	224	NA
Mean intron length	526 bp	391 bp	716	NA
Mean number of exons per gene	6.1	6.4	8	NA

*Note*: ^a^ The genome of *H. robusta* (Ref7), *W. pigra* (this study), *H. medicinalis* (Ref8), *H. medicinalis* (Ref9) were annotated using Genewise, BRAKER_v2, AUGUSTUS_v3 and MAKER_v2, respectively. NA, the data is not available in the references. GC, fraction of guanine plus cytosine nucleobases. Scaffold N50, the length such that half of the assembled sequence is in scaffolds longer than this length

### Syntenic blocks among the genomes of *W. pigra, H. medicinalis* and *H. robusta*

**Fig. 2:**
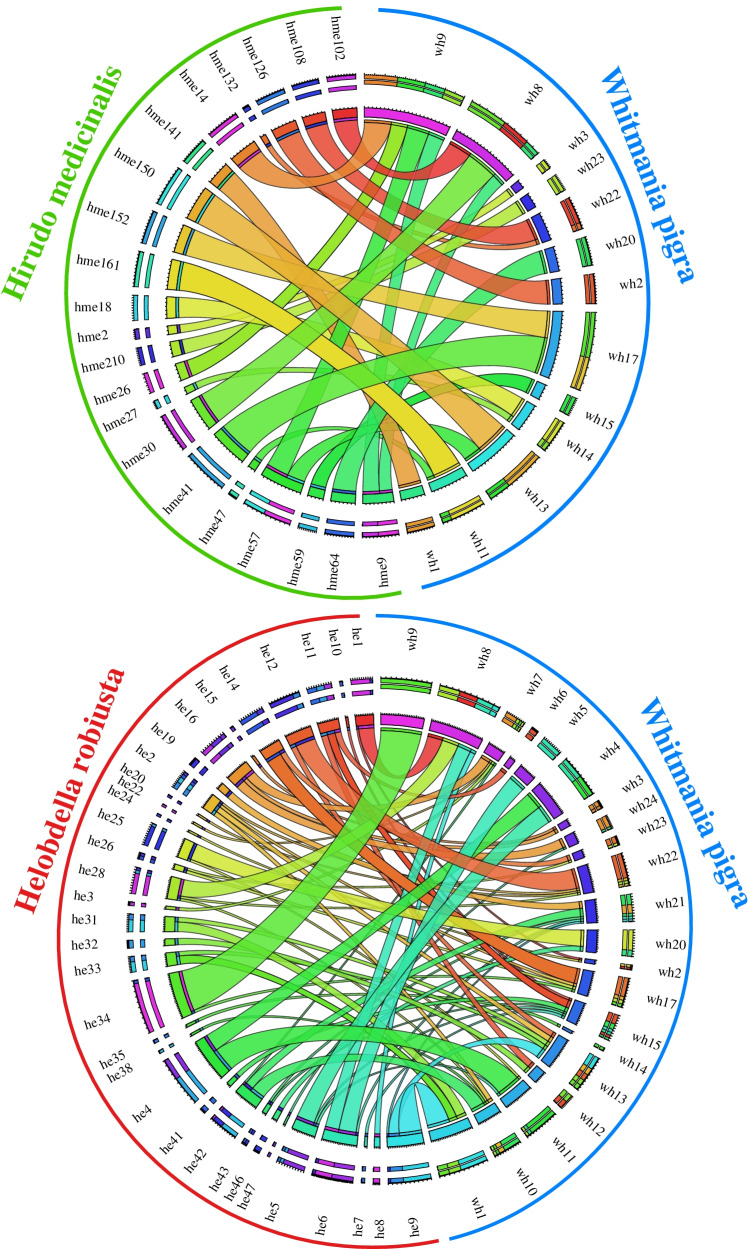
Syntenic relationships between *W. pigra* and *H. medicinalis*, and between *W. pigra* and *H. robusta*. The top panel represents the syntenic relationships between *W. pigra* and *H. medicinalis.* The bottom panel shows the syntenic relationships between *W. pigra* and *H. robusta.* The scaffolds will be connected if they share similar genes. The width of link represents the number of shared genes

The above result showed that *W. pigra* only has 46.2% (12346) orthologous genes in *H. medicinalis, and* 38.4% (10295) orthologous genes in *H. robusta*. To further compare the genome similarity among the three leech species, we performed a careful analysis of syntenic blocks between *W. pigra* and *H. medicinalis,* and between *W. pigra* and *H. robusta* using MCScanX [15]. As small scaffolds are not useful for gene synteny analysis, we only considered the scaffold with more than 30 genes. A total of 25, 48, and 47 scaffolds for *W. pigra*, *H. medicinalis*, and *H. robusta*, respectively, were used to find syntenic blocks using MCScanX. Finally, we identified 21 scaffolds in *H. medicinalis* with syntenic blocks matched to the 13 scaffolds in *W. pigra*. In contrast, there are 33 scaffolds in *H. robusta* matched to the 21 scaffolds in *W. pigra* (Fig. 2). Overall, the genome of *W. pigra* has a good collinearity relationship with the other two genomes. We further examined the synthetic blocks in the larger scaffolds wh8, wh9, wh17, and wh22. We found that *H. medicinalis* tends to have larger synthetic blocks matched to the scaffolds of *W. pigra* than *H. robusta.* It suggests that compared to *H. robusta, W. pigra* has a more similar genome structure to *H. medicinalis.*

### The expansion and contraction of gene family in the *W. pigra* genome

**Fig. 3:**
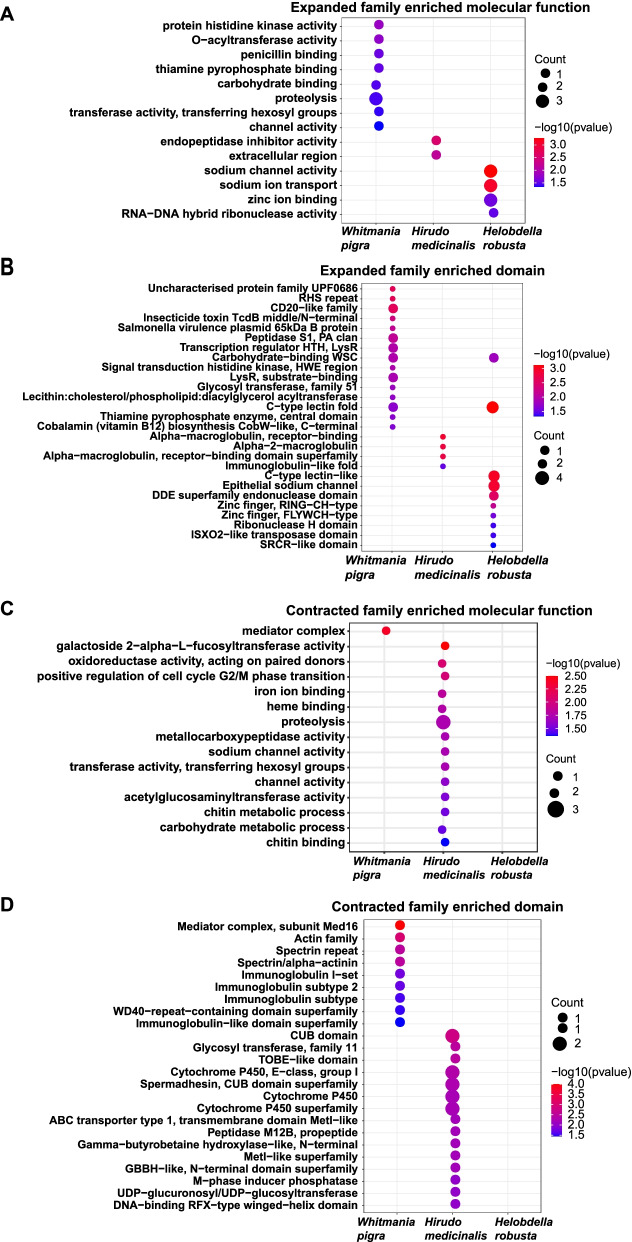
Enrichment analysis of expanded and contracted gene families between the three species using GO terms and interPro domains. GO terms (**A**) and interPro domains (**B**) were enriched by expanded gene families in *W. pigra*, *H. medicinalis* and *H. robusta*; GO terms (**C**) and interPro domains (**D**) were enriched by contracted gene families in *W. pigra*, *H. medicinalis* and *H. robusta*

After analysis of gene synteny, we further analyzed the expansion and contraction of gene family among the seven species: *H. robusta*, *Lottia gigantea*, *Capitella teleta*, *Schmidtea mediterranea*, *Schistosoma mansoni*, *W. pigra, H. medicinalis*. We first compared the predicted proteomes of seven species using OrthoFinder [16], yielding a total of 13563 orthologous gene families that comprised 108245 genes. The gene families and their numbers of members for the seven species were supplied to the software package CAFE (v4.1) [17]. Then CAFE applied the likelihood model to identify the expanded and contracted gene family along each branch of the phylogenetic tree. Finally, we found 1488, 832 and 1266 gene families expanded in *W. pigra, H. medicinalis* and *H. robusta*, respectively (Fig. 1D). Of these families, there are 63, 1 and 59 families that are evolving rapidly (P<0.05) in *W. pigra, H. medicinalis* and *H. robusta*, respectively (Fig. 1E). These rapidly evolving families are species-specific and little overlap between the two species (Fig. 1E). To reveal the molecular function and structural domain of these rapidly evolving families, we performed enrichment analyses by gene ontology terms and interPro domains. The enrichment results showed a clear difference among the three leech species. For *W. pigra*, the expanded families are enriched in the following functions: protein histidine kinase activity, O−acyltransferase activity, thiamine pyrophosphate binding, carbohydrate binding, proteolysis, etc. For *H. robusta*, the expanded families are mainly enriched in functions such as sodium channel activity, sodium ion transport, zinc ion binding, and RNA−DNA hybrid ribonuclease activity. For *H. medicinalis,* only two functions endopeptidase inhibitor activity and extracellular region are enriched (Fig. 3A). In contrast, for the contracted families, there are little go terms enriched in *W. pigra* and *H. robusta*, but more go terms enriched *in H. medicinalis.* For example, iron ion binding, heme binding, proteolysis, and sodium channel activity functions are enriched by the contracted family in *H. medicinalis* (Fig. 3C)*.* Corresponding to these functions, specific protein domains are enriched in different leeches (Fig. 3B and D). These results imply the three species may take different adaptive strategies. And the different functions and domains are potentially related to environmental adaptation and bioactive peptides properties of the three leech species.

### Phylogenetic analysis and sequence alignment of the hirudin gene family

**Fig. 4:**
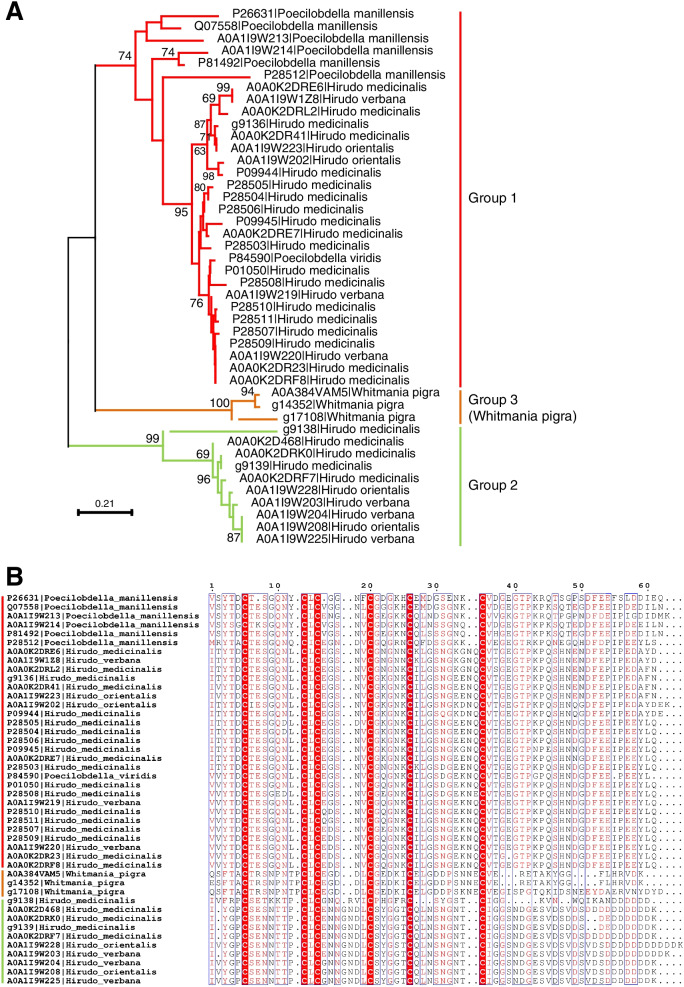
Sequence analysis of the hirudin gene family. **A** Phylogenetic analysis of hirudin gene family from the species of family *Hirudinidae*. The tree was inferred by using the Maximum Likelihood method and JTT matrix-based model Likelihood method. **B** Multiple alignments of the amino acid sequences of hirudin proteins

**Fig. 5:**
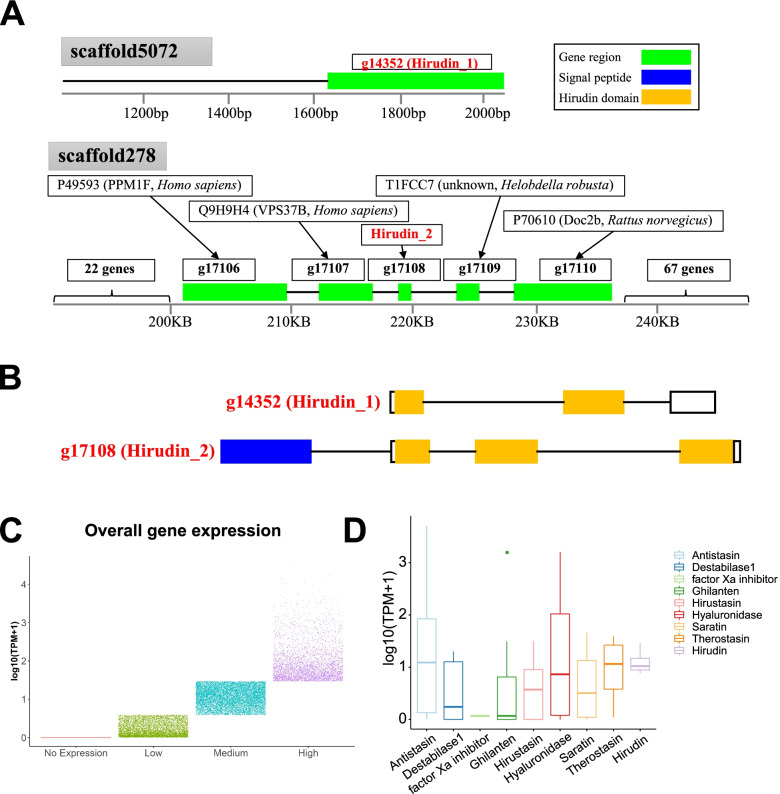
Detailed analysis of hirudin genes and gene expression in *W. pigra*. **A** Genome-wide distribution of hirudin genes; **B** Gene structure of hirudin genes; **C** Jitter plot shown overall gene expression in *W. pigra*. **D** Gene expression of different classes of bioactive peptides

As the most well-studied natural anticoagulant from leeches, hirudin has served as a standard for designing natural coagulation inhibitors [18]. Hirudin may be useful in the therapy of thrombosis because of its specific antithrombin effects [19]. We identified two hirudin genes g14352 and g17108 (Fig. 4A) in *W. pigra* in this study. We named g14352 and g17108 as hirudin_1, hirudin_2, respectively (Fig. 5). For comparison, we also identified three hirudin genes g9136, g9138, and g9139 in *H. medicinalis.* These five hirudin genes and 38 hirudin-like sequences from protein database UniProt were used to clarify the phylogenetic relationships of these hirudin genes (Fig. 4A). They are clustered into three clades (named Groups 1, 2 and 3) (Fig. 4A). Three groups are highly supported with bootstrap value (74, 99, and 100, for Groups 1, 2 and 3, respectively). The sequences (Group 3) from *W. pigra* do not cluster with the other hirudin genes. Groups 1, 2 and 3 follow different cysteine patterns CX(7)CX(1)CX(5)CX(5)CX(10)C, CX(7)CX(1)CX(5)CX(5)CX(8)C, and CX(8)CX(1)CX(5)CX(5)CX(10)C, respectively (Fig. 4B). The pattern of group 1 is the typical cysteine pattern of the hirudin. In contrast, gene g17108 (hirudin_2) of *W. pigra* shows the third cysteine pattern, which inserts an extra amino acid between the first and second cysteines. In addition, we have calculated the pairwise similarities among the hirudin sequences (Fig. S2). Within the group, the pairwise similarities of hirudins are more than 60%. In different groups, the pairwise similarities of hirudins are between 30 and 60%. Whether between G2 and G1 or between G3 and G1, the pairwise similarities are always below 60%. The gene g17108 (hirudin_2) is a new kind of hirudin, which has not been reported before. The actual function of the three hirudin in *W. pigra* deserves further experiment investigation.

### Genome-wide distribution and gene structure of hirudin genes

Although there are a lot of studies about hirudin, the genome-wide distribution and gene structure of hirudin have not been reported. By sequence searching, we found that g14352 (hirudin_1) and g17108 (hirudin_2) are located at different scaffolds 5072 and 278, respectively (Fig. 5A). The left and right sides of g17108 (hirudin_2) are surrounded by multiple genes (24 and 69 genes, respectively). We can infer that the two genes are separated by great distances (>210 Kbp). It suggested a lot of genome rearrangement events happened after gene duplication of hirudin genes. Furthermore, gene structures of the two hirudin genes are also different. g14352 (hirudin_1) only has three exons. In contrast, g17108 (hirudin_2) has four exons, which encode a signal peptide and a longer tail. Therefore, protein hirudin_2 has a longer sequence than hirudin_1 (Fig. 5B).

### Exploration and gene expression of bioactive ingredients in the *W. pigra* genome

There are more than 20 bioactive substances identified from leeches, such as Antistasin, hirustasin, ghilantens, hirudin [2, 6, 20, 21]. These molecules have analgesic, anti-inflammatory, anticoagulant, platelet inhibitory, thrombin regulatory functions, and so on. *W. pigra* and *H. medicinalis* belong to the family *Hirudinidae*. In contrast, *H. robusta* belongs to the family *Glossiphoniidae*. The detailed gene copies of these bioactive substances in different leech species is still unknown. It is essential to identify and compare these active molecules in different leeches. Using genome data, we systematic explored and compared five classes of active substances in *W. pigra, H. medicinalis* and *H. robusta* (Table 2)*.* All 9 common active molecules were found in *W. pigra*. It is noteworthy that hirustasin, hirudin and destabilase I genes are absent in the *H. robusta.* There are far more gene copies for the active molecules in *W. pigra* than in *H. robusta* (57 vs 24). *W. pigra* exceeds *H. robusta* in both kinds and gene number of active molecules. The gene copy of bioactive ingredients of *W. pigra* also exceeds that of *H. medicinalis*. To make full use of the available RNA-seq data, we examined the gene expression of bioactive peptide in *W. pigra* with the overall gene expression as reference. We divided all genes of *W. pigra* into four parts (No expression, Low, Medium, High). The expression of these genes is shown in Fig. 5C. All kinds of bioactive peptides were expressed (Fig. 5D). Of these peptides, antistasin, therostasin, and hirudin have higher expression, while factor Xa inhibitor and ghilanten have lower expression. The result implies that these bioactive peptides may play different roles in the survival of *W. pigra*.

**Table 2:** The exploration of five class of active substances in *W. pigra, H. medicinalis* and *H. robusta*

Modes of action	Bioactive molecules	Gene copy (***H. robusta)***	Gene copy (***W. pigra)***	Gene copy (***H. medicinalis)***
Analgesic and anti-inflammatory effect	Antistasin	6	9	5
	Hirustasin	0	18	7
	Ghilanten	9	10	7
Extracellular matrix degradation	Hyaluronidase	1	4	5
Inhibition of platelet function	Saratin	1	4	1
Anticoagulant effect	hirudin	0	2	3
	Factor Xa inhibitor	2	1	1
	Therostasin	5	4	2
	Destabilase I ^a^	0	5	2
Antimicrobial effect	Destabilase I ^a^	0	5	2
Total		24	57	33

^a^ Destabilase I, involved in anticoagulant effect and antimicrobial effect

## Discussion

“Medicinal leech” represents the leeches in the family *Hirudinidae* of the order hirudinida. Medicinal leeches have been widely utilized in medical procedures for thousands of years and were approved by the US Food and Drug Administration in June, 2004 as a medical device due to their mechanically relieving venous congestion and delivering anti-coagulants [22, 23]. *W. pigra* is the most commonly available from Chinese commercial leech market. Although its importance in medicine and the significance of medicinal leeches in biological research, there is no genome data available for any species in the family *Hirudinidae* until 2020. The genome of *H. robusta* has been sequenced to study bilaterian evolution in 2013. *H. robusta* is a leech of the family *Glossiphoniidae*, which is very far from the family *Hirudinidae* [24]. The genome of *H. medicinalis* in the family *Hirudinidae* has been reported recently and studied from different perspectives [8, 9]. In this study, we reported the genome of *W. pigra,* another medicinal leech in the family *Hirudinidae*. We characterized the genome by analysis of gene synteny, gene family and the gene copies of bioactive molecules and comparing them with *H. robusta* and *H. medicinalis*.

The results of the expansion and contraction of gene family revealed very clear different patterns among *W. pigra*, *H. medicinalis* and *H. robusta*. This suggests that the three leech species used different survival strategies to adapt to living environment. These results also suggest that although *W. pigra* and *H. medicinalis* both are medical leeches, they displayed different patterns of expanded and contracted families. Therefore, the features of one leech species cannot simply be applied to another leech species. Specially must point out here, while *W. pigra* and *H. medicinalis* both are used medicinally, the two species have very different feeding habits. *H. medicinalis* is sanguivorous by its two suckers. In contract, *W. pigra* is predatory and feeds primarily by swallowing other invertebrates. Therefore, all differences between *W. pigra* and *H. medicinalis* are due to the selection pressure of survival, not that humans used them for medicine. The details of how the selection pressure shapes the difference of gene family deserve further investigation.

In respect of active substances, we found a huge difference between *W. pigra* and *H. robusta* after systematic comparison of five classes of active substances. Hirudin, hirustasin, and destabilase I genes are absent in *H. robusta*. In contrast, all nine common active molecules were found in *W. pigra*. There are two hirudin genes in *W. pigra*. Furthermore, two hirudin genes display different cysteine patterns in the protein sequence. The gene g17108 (hirudin_2) is a new kind of hirudin, which has not been reported before. It remains unknown that the detailed role of anticoagulant peptides in the life history of *W. pigra*, as predatory animals. The comparison of this gene g17108 (hirudin_2) with all available hirudin sequence may provide insight into the development of a new hirudin with more potent activity. Significantly, although *W. pigra* and *H. medicinalis* are both medicinal leeches, the gene copy of bioactive ingredients of *W. pigra* far exceeds that of *H. medicinalis*. It is worth noting that the difference in gene copies between the two species may partly be due to the usage of different gene annotation tools. The genome of *H. robusta* [7], *W. pigra* (this study), *H. medicinalis* [8], *H. medicinalis* [9] were annotated using Genewise, BRAKER_v2, AUGUSTUS_v3 and MAKER_v2, respectively. Ideally, all comparisons should be done with uniform standards and methods.

In the recent studies of *H. medicinalis* genome, both Babenko VV and Kvist S explored the bioactive peptides in *H. medicinalis.* The study of Kvist S focused on the 18 well-characterized leech-derived proteins related to antihemostasis. While Babenko VV performed proteomic analysis and compared salivary cell secretions from three medicinal leech species, *H. medicinalis*, *H. orientalis*, and *H. verbena*. They described and analyzed the enzymes including proteases, superoxide dismutase, hyaluronidase, etc., proteinase inhibitors and molecules involved in adhesion. Our study explored nine classes of bioactive peptides with five biological effects such as analgesic and anti-inflammatory effect, extracellular matrix degradation, inhibition of platelet function, anticoagulant effect and antimicrobial effect (Table 2). Our result is similar to the result of Kivst et al and Babenko VV et al. This study identified three hirudin genes g9136 (Group1), g9138 (Group2), and g9139 (Group2) in the genome of *H. medicinalis*, which are the same in the study of Babenko VV et al. However, only two genes g9136 (Group1) and g9139 (Group2) were identified by Kivst et al. The author explained that miss genes may be present in the unsequenced parts of the genome. Many other peptides are not covered simultaneously by all three studies. All bioactive peptides mentioned by this study and the other studies deserve further investigation with uniform standards and methods.

## Conclusions

In summary, the genome of another medicinal leech (*W. pigra*) was reported in this study. The genomes of three leech species, *W. pigra, H. medicinalis* and *H. robusta,* show many differences in the respects of orthologous genes, gene synteny and gene family. Furthermore, *W. pigra* exceeds *H. robusta* in both kind and gene number of active substances, such as hirudin, hirustasin, and destabilase I genes. This study pointed out the differences in the genome of two medicinal leeches, *W. pigra* and *H. medicinalis* and provided insight into the exploration and development of bioactive molecules of medicinal leeches.

## Methods

### Sample preparation and genome and RNA sequencing

A total of seven samples of *W. pigra* were collected from East Lake in Wuhan, China (GPS Coordinates: E99°17′23.62″, N25°12′68.55″). Animal care and handling were conducted in accordance with the stipulations of Ethics Committee of Kunming University. Genomic DNA was extracted from the whole body of one single sample without gastric tracts and blood. Two short paired-end (300 and 500 bp) and three mate-end (5, 8, and 10 Kbp, respectively) sequencing libraries were constructed with the standard protocol provided by Illumina (San Diego, United States), and then sequenced on an Illumina HiSeq platform. Low-quality and duplicated reads were filtered out through fastp (v0.20.0) software [25].

For RNA-seq, RNA extraction and sequencing were performed as previously described [13]. Briefly, tissues cleaned off gastric tracts and the blood were preserved in liquid nitrogen for RNA extraction. Total RNAs were purified with RNA Easy Kit (QIANGEN, German). Total RNA yields and the quality were measured by agarose gel electrophoresis and spectrophotometer (Thermo, USA). And mRNA was isolated with Oligo-dT Purist Kit (TaKaRa, Japan). The Illumina TruSeq RNA sample preparation kit (San Diego, United States) was used to prepare the library. Then the library was sequenced by Illumina HiSeq platform at Biomarker Technologies company in China.

### Estimation of genome sizes and genome assembly

Genome sizes were estimated using JELLYFISH [26] and GenomeScope [27] with an optimal k-mer size (K-mer=23). Genome sizes were calculated from the following equation: Genome size = 23-mer_number / 23-mer_depth, where 23-mer_number is the total number of each unique 23-mer and 23-mer depth is the highest frequency that occurred. Consequently, the estimated genome size of *W. pigra* was ~ 162 Mbp. By taking the estimated genome size as a reference, total sequence data accounted for ~100-fold coverage. The clean reads were used for de novo assembly by Platanus (v1.2.4) [28] with default parameters. Subsequently, intra-scaffold gaps were filled using the reads of short-insert libraries by gap_close command. The final assembled genome size was ~ 177 Mbp. The summary for assembly results is list in Table 1. Only scaffolds with lengths longer than 500 bp were used in further analyses.

### Genome annotation

Homolog and de novo strategies were both applied to identify the repetitive sequence in the *W. pigra* genome. Software LTRfinder (v1.07) [29] and RepeatModeler (v1.0.11, http://www.repeatmasker.org/RepeatModeler) were used for ab initio prediction. The results obtained from these tools were combined to form a new repetitive sequence database. This database was then merged with Repbase [30, 31]. Repetitive sequences in the *W. pigra* genome were identified by homolog searching with the final merged database by RepeatMasker (v1.332) [32]. We identified 40 Mbp repetitive sequences, which accounted for 23% of the *W. pigra* assembled genome (Table 1). Protein coding genes were predicted using GeneMark-ES (v4.3.8) and AUGUSTUS (v3.3.0) implemented in the BRAKER2 pipeline [33, 34] using RNA-seq alignments as evidence. The RNA-seq bam files generated by HISAT2 [35, 36] were combined and fed into BRAKER. A total of 26,743 protein-coding genes were generated for the *W. pigra* genome.

All protein sequences from the BRAKER2 results were aligned to TrEMBL and UniProt [37] databases using BlastP at E-value ≤1e^-5^. Gene functions were also annotated using the InterProScan software [38–40] by searching publically available databases including Pfam [41, 42], PRINTS [43], ProDom [44] and SMART [45]. In summary, approximately 95% (25,496/26,743) of the genes were supported by at least one related function assignments from the public databases (TrEMBL, UniProt, and InterPro).

### Comparative genomic analysis

To define gene families that descended from a single gene in the last common ancestor, we downloaded the protein-coding genes of *H. robusta, Lottia gigantea, Capitella teleta, Schmidtea mediterranea, Schistosoma mansoni* from NCBI. The protein-coding genes of *H. medicinalis* were downloaded from http://download.ripcm.com/hirudo_genome. The protein-coding genes of *W. pigra* were derived from BRAKER2. All proteins of the seven species were processed with OrthoFinder-Diamond (v1.1.10) to provide information about orthologous gene families. OrthoFinder is robust to incomplete models, differing gene lengths, and larger phylogenetic distances [16]. Gene families (orthogroups) in OrthoFinder are defined as homologous genes descended from a single gene from the last common ancestor of the species examined. It is assumed that a parental gene of each orthogroup was present in the common ancestor of the seven species investigated. We applied the likelihood model implemented in the software package CAFE (v4.1) [17] to identify the expanded and contracted gene family along each branch of the phylogenetic tree. The phylogenetic tree was constructed in the process of defining gene families. The syntenic blocks based on the protein-coding genes locations in the genome were calculated by software MCScanX with default parameters.

### Phylogenetic analysis of gene family

Protein sequences in the gene family were aligned using Clustal W [46] with fine adjustment by hand. Then the aligned sequences were used for phylogenetic analysis using MEGA X [47]. The evolutionary history was inferred by using the Maximum Likelihood method and JTT matrix-based model [48]. The percentage of trees in which the associated taxa clustered together is shown next to the branches. Initial tree(s) for the heuristic search were obtained automatically by applying Neighbor-Join and BioNJ algorithms to a matrix of pairwise distances estimated using a JTT model, and then selecting the topology with superior log likelihood value. The tree is drawn to scale, with branch lengths measured in the number of substitutions per site. The default parameters were used for sequence alignment, phylogenetic analysis.

## Supplementary Information

**Additional file 1: Supplementary figure S1 and S2. Figure S1**. GenomeScope Analysis of the 23-mers for W. Pigra genome sequencing data. Estimate of the heterozygous portion is 0.765%. The estimated genome size of W. Pigra was ~ 162 Mbp. **Figure S2**. The heatmap displays the pairwise similarities values among the Hirudin sequences in Fig. 4. The Hirudin sequences belong to G1, G2, G3, respectively. Within the group, the pairwise similarities of Hirudins are more than 60%. In contrast, in different groups, the pairwise similarities of Hirudins are between 30 and 60%. Whether between G2 and G1 or between G3 and G1, the pairwise similarities are always below 60%.

## Data Availability

All of the raw data generated in this study have been deposited in the National Center for Biotechnology Information (NCBI) [49] under BioProject accession PRJNA792259 that is publicly accessible at https://www.ncbi.nlm.nih.gov/bioproject/PRJNA792259/. All other data are available from the authors upon reasonable request.

## Abbreviations

*W. pigra*: *Whitmania pigra*
*H. medicinalis*: *Hirudo medicinalis*
*H. robusta*: *Helobdella robusta*
RBHs: Reciprocal best blast hits
