# Supplementary material for: The genome of medicinal leech (Whitmania pigra) and comparative genomic study for exploration of bioactive ingredients
Source: BMC Genomics. 2022 Jan 24;23:76. doi: 10.1186/s12864-022-08290-5 (PMC8787918; doi:10.1186/s12864-022-08290-5)
Supplement: Supplementary file 1 — Additional file 1: Supplementary figure S1 and S2. Figure S1. GenomeScope Analysis of the 23-mers for W. Pigra genome sequencing data. Estimate of the heterozygous portion is 0.765%. The estimated genome size of W. Pigra was ~ 162 Mbp. Figure S2. The heatmap displays the pairwise similarities values among the Hirudin sequences in Fig. 4. The Hirudin sequences belong to G1, G2, G3, respectively. Within the group, the pairwise similarities of Hirudins are more than 60%. In contrast, in different groups, the pairwise similarities of Hirudins are between 30 and 60%. Whether between G2 and G1 or between G3 and G1, the pairwise similarities are always below 60%. [file 12864_2022_8290_MOESM1_ESM.pdf]

Supplementary information

The genome of medicinal leech (*Whitmania pigra*) and comparative genomic study for exploration of bioactive ingredients

Lei Tong[1, #], Shao-Xing Dai[2, #], De-Jun Kong[1, #], Peng-Peng Yang[2], Xin Tong[2], Xiang-Rong Tong[1], Xiao-Xu Bi[1], Yuan Su[1], Yu-Qi Zhao[3], Zi-Chao Liu[1, *]

[1]Engineering Research Center for Exploitation and Utilization of Leech Resources in Universities of Yunnan Province, School of Agronomy and Life Sciences, Kunming University, Kunming 650214, China

[2]State Key Laboratory of Primate Biomedicine Research, Institute of Primate Translational Medicine, Kunming University of Science and Technology, Kunming 650500, China

[3]Department of Integrative Biology and Physiology, University of California, Los Angeles, Los Angeles, CA 90095, USA

Authors email:

Lei Tong: 1501159559@qq.com

Shao-Xing Dai: daishx08@gmail.com

De-Jun Kong: kongdj05@hotmail.com

Peng-Peng Yang: yangpp@lpbr.cn

Xin Tong: tongxin0013@163.com

Xiang-Rong Tong: txiangrong@163.com

Xiao-Xu Bi: 279781667@qq.com

Yuan Su: suyuankm@163.com

Yu-Qi Zhao: zhaoyuqi616@gmail.com

Zi-Chao Liu: abclzc@aliyun.com

#These authors contributed equally to this work

*Corresponding author: Zi-Chao Liu

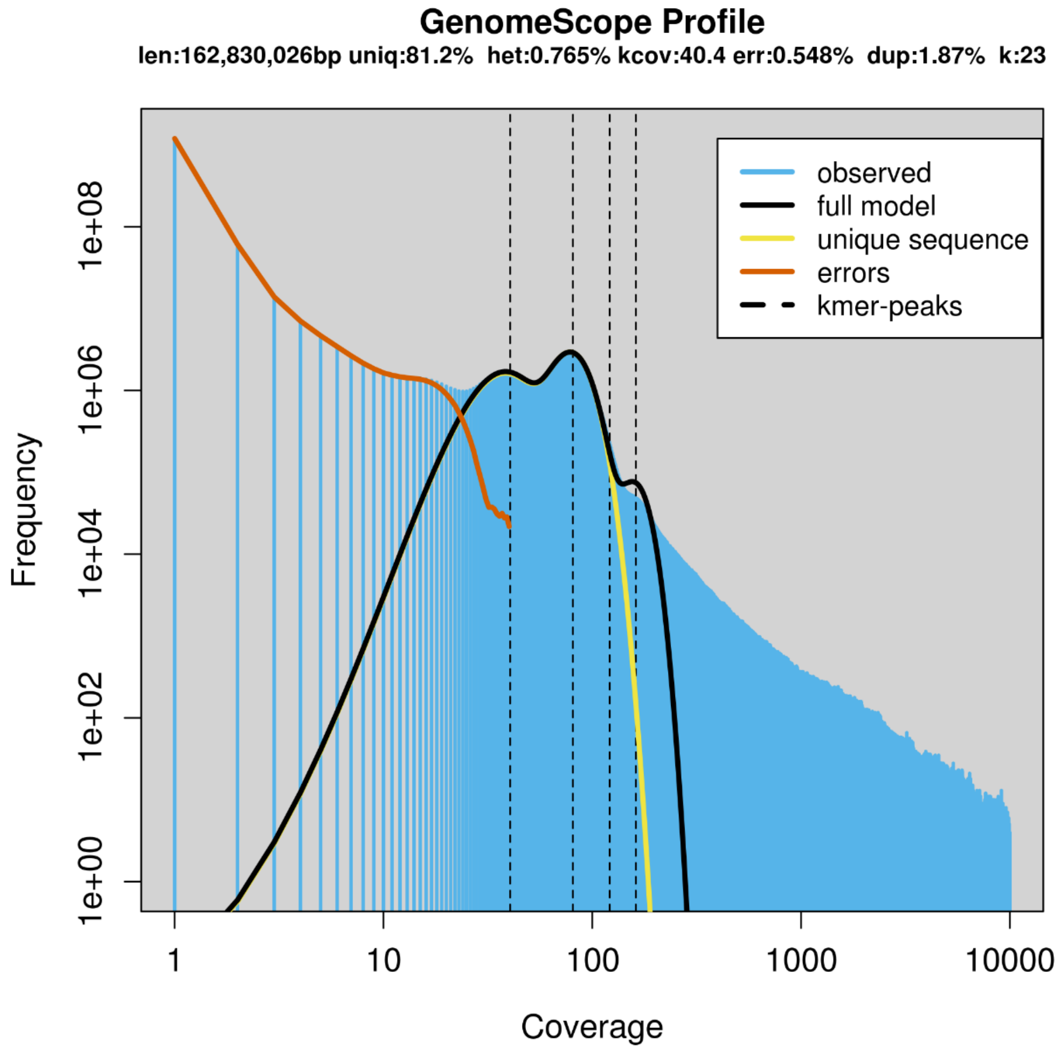

Figure S1. GenomeScope Analysis of the 23-mers for W. Pigra genome sequencing data. Estimate of the heterozygous portion is 0.765%. The estimated genome size of W. Pigra was ~ 162 Mbp

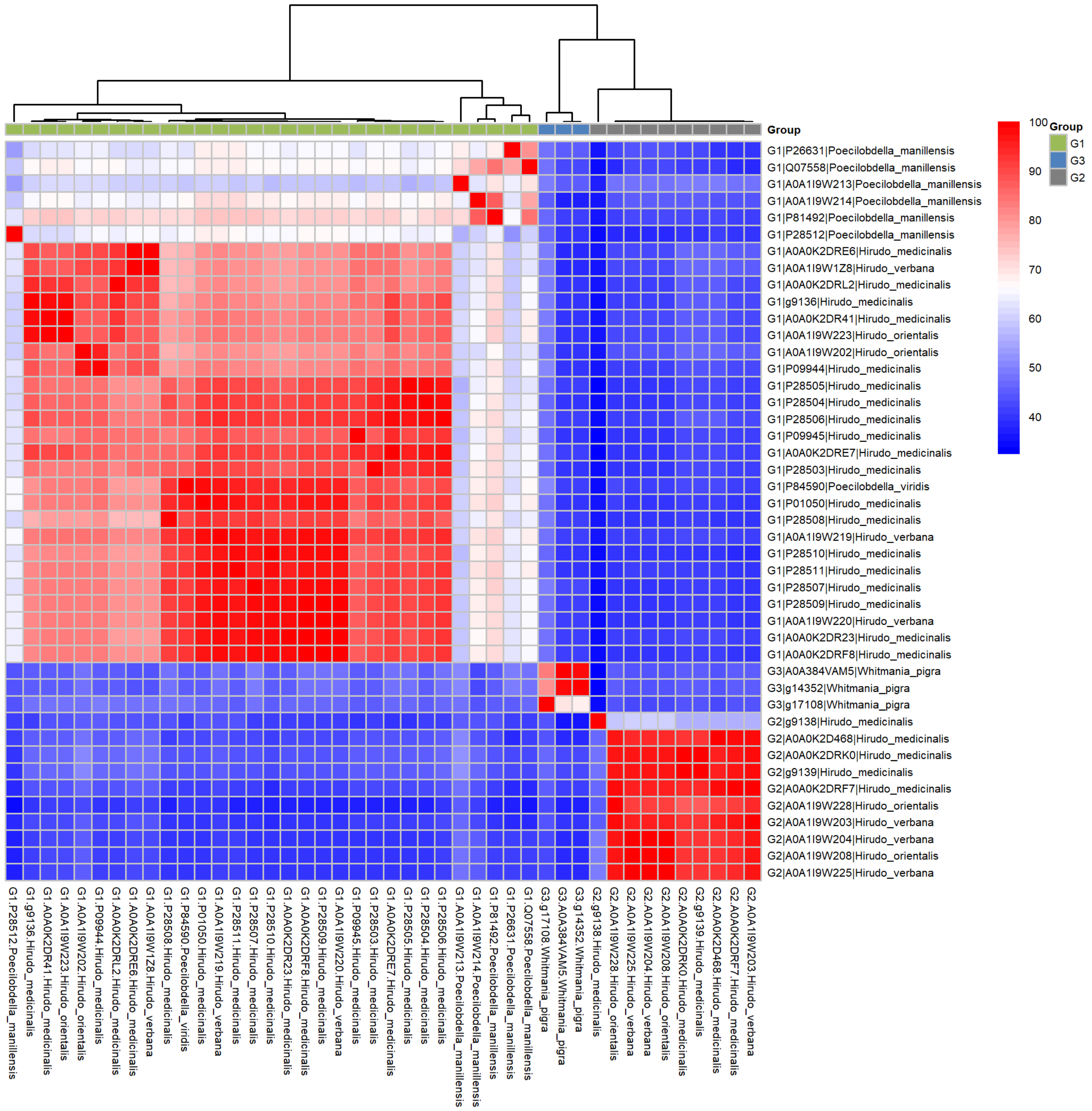

Figure S2. The heatmap displays the pairwise similarities values among the Hirudin sequences in **Figure 4**. The Hirudin sequences belong to G1, G2, G3, respectively. Within the group, the pairwise similarities of Hirudins are more than 60%. In contrast, in different groups, the pairwise similarities of Hirudins are between 30% and 60%. Whether between G2 and G1 or between G3 and G1, the pairwise similarities are always below 60%.
